# 2511: Use of an online provider learning community to assess clinical HIV/HCV/STDs-related training needs

**DOI:** 10.1017/cts.2017.182

**Published:** 2018-05-10

**Authors:** Cabiria Monica Barbosu, Jose G. Perez-Ramos, Margaret Demment, Thomas Fogg, Jack Chang, Beatrice Aladin, Cheryl Smith, Timothy De Ver Dye, Terry Doll

**Affiliations:** University of Rochester Medical Center, Rochester, NY, USA

## Abstract

OBJECTIVES/SPECIFIC AIMS: The prevention, management, and treatment of HIV, STDs, and HCV requires continuous training that reflects contemporary best-practice and innovative care models. In order to improve the NYS AIDS Institute’s comprehensive web-enabled training program, which enhances the capacity of a diverse healthcare workforce, a needs assessment (NA) of our community of practice (CoP) is needed to better understand their training needs, circumstances, and instructional modalities preferences. The goal of the assessment was to better understand our CoP’s preferences of online trainings, and as a result to develop a “responsive design” system that will enhance user’s learning experience thus improving patient care. METHODS/STUDY POPULATION: We developed and deployed an NA survey using REDCap. The instrument consisted in 27 questions related to providers’ preferences on receiving continuing educational training and their use of technologies, including mobile platforms, online modules, webinars, and telehealth. As part of the recruitment strategy, several resources were deployed over a 1-month recruitment period including sequential email blasts, website promotion, and assessment links included in newsletters and social media. Weekly reminders were also used to promote the participation from our CoP. RESULTS/ANTICIPATED RESULTS: A total of 310 respondents participated in the NA, with 85.8% from NYS. 177 were clinicians (20.5% MD, 2.9% PA, 17.3% NP, and 16.3% RN) and 133 nonclinical providers (case/care managers, social workers, public health professionals, coordinators/administrators, and other). The participants worked in hospitals, community health centers, substance use centers, private practices, and state/local health departments. More than 90% of respondents indicated that they preferred both live/in-person and online training, and participants most strongly indicated that they stayed up-to-date on current developments through CDC, the AIDS Institute, and conferences. More than 60% of respondents considered that receiving CE credit for the training was very important and 28% indicated they would use training materials in Spanish if offered. In terms of technology, over 80% of the respondents preferred computers, but more 50% also used mobile devices (computer at home 61.8%, computer at work 85%, tablet 29.9%, iPhone 20.9%, Android 16.6%, other device 2.3%). DISCUSSION/SIGNIFICANCE OF IMPACT: Accessing an online CoP provided a useful opportunity to assess training needs and preferences of clinical and nonclinical providers. Most providers indicated that they were primarily likely to use a work computer to complete online training or secondarily a home computer. With a significant portion of respondents indicating use of tablets, smartphones, and other devices, online training opportunities should be developed with responsive design to assure flexibility and access. In addition to online training, participants indicated that they also strongly valued live, in-person training. Offering training with CDC and the NYS AIDS Institute branding, in Spanish, together with offering continuing education credit, were all seen as desirable training elements. Accessing this online CoP helped streamline and target training priorities and logistics.

